# Contrast-enhanced transcranial doppler versus contrast transthoracic echocardiography for right-to-left shunt diagnosis

**DOI:** 10.1007/s10877-023-00979-6

**Published:** 2023-02-18

**Authors:** Li Tian, Min Zhang, Hongjun Nie, Guanling Zhang, Xiaoyan Luo, Huaiyun Yuan

**Affiliations:** Department of Ultrasound Diagnostics, The Affiliated Changsha Central Hospital, Hengyang Medical School, University of South China, 161 Shaoshan South Road, Yuhua District, 410004 Changsha, Hunan People’s Republic of China

**Keywords:** Contrast-enhanced transcranial Doppler, Contrast transthoracic echocardiography, Right-to-left shunt, Patent foramen ovale, Transesophageal echocardiography

## Abstract

RLS can be diagnosed using US, CT angiography, and right heart catheterization. However, most reliable diagnostic modality remains undetermined. c-TCD was more sensitive than c-TTE in the diagnosis of RLS. This was true especially for the detection of provoked shunts or mild shunts. c-TCD can be used as the preferred screening method for RLS.

## Introduction

The prevalence of primary headache is high in the general population, imposing a significant burden on society and individuals [1]. Numerous studies have shown that migraine may be associated with a right-to-left shunt (RLS) [2–4], which is an abnormal pathway in the arteriovenous system. When the pressure of the right cardiac system is higher than that of the left cardiac system, microemboli and high concentrations of metabolites such as serotonin in the right cardiac system can enter the left cardiac system through this abnormal pathway, leading to diseases such as migraine and cryptogenic stroke [5]. This abnormal shunt may present as either intracardiac or extracardiac shunt. Patent foramen ovale (PFO) is the most common type of cardiac shunt, accounting for approximately 95% of all circulatory RLSs. Extracardiac shunts include pulmonary arteriovenous malformation and patent ductus arteriosus, among others [6, 7].

RLS can be diagnosed using ultrasonography, computed tomography angiography, and right heart catheterization [2]. Many studies have used ultrasound to diagnose RLS [2, 8–10], including contrast-enhanced transcranial Doppler (c-TCD), contrast transthoracic echocardiography (c-TTE), and transesophageal echocardiography (TEE) with or without contrast. However, which examination method is most reliable remains controversial.

c-TCD is a noninvasive, safe, and comfortable examination method with no obvious side effects. It has been reported that c-TCD has high sensitivity and specificity for the diagnosis of RLS, and its accuracy is higher than that of c-TTE and contrast transesophageal echocardiography (c-TEE). Therefore, c-TCD could be used as the preferred screening method for RLS [9, 11, 12]. However, this imaging modality has certain limitations, one of which is acoustic window limitation, especially in older patients. When the acoustic window is limited, the vertebral artery or the internal carotid artery can be used instead of the middle cerebral artery to monitor the microbubble signal [13]. Another limitation of c-TCD is that it cannot distinguish between intracardiac and extracardiac shunts; however, the addition of c-TTE to the c-TCD imaging protocol can improve its specificity [10]. Chen et al. believed that both c-TCD and c-TTE have high sensitivity and that there is no significant difference in sensitivity [8]. Liu et al. found that c-TTE with the Valsalva maneuver had higher sensitivity [2]. Yang et al. also found that c-TEE would not only have false negative results, but also underestimated the shunts in 44% of patients presenting with larger shunts, as determined by c-TTE [9]. Retrospective analysis and comparison of c-TCD and c-TTE examination was the first aim of this study. The uniqueness of this study lay in the use of large sample data to further confirm the advantages and limitations of each method.

## Materials and methods

### Study design and procedures

A retrospective study was conducted involving patients and healthy volunteers who underwent RLS screening at the Affiliated Changsha Central Hospital, Hengyang Medical School, University of South China, Hunan, China, from June 2015 to December 2021. The inclusion criteria were as follows: (1) complete head computed tomography or magnetic resonance imaging examination; (2) complete examination with TCD and c-TCD; and (3) follow-up completion with TTE, c-TTE, or TEE. The exclusion criteria were as follows: (1) unable to complete the Valsalva maneuver, (2) consciousness disorder caused by severe cerebral infarction or cerebral hemorrhage, (3) extensive abnormal intracranial blood flow, (4) recent infection and thrombosis, and (5) severe cardiopulmonary insufficiency. All patients and healthy volunteers signed informed consent forms before the c-TCD, c-TTE, and TEE examinations. The patients’ basic information, test results, and clinical diagnosis information were collected. This study was approved by the institutional review board of Changsha Central Hospital. All procedures were performed in accordance with the principles of the Declaration of Helsinki.

### Inspection method

Contrast media configuration: After placing a 18G trocar in the right cubital vein of the patient, connected to a three-way tube and a 10 ml syringe (with 9 ml of normal saline and 1 ml of air and 1 drop of the patient venous blood). Activated saline was formed after vigorous exchange of mixed saline and air between the two syringes 30 times [6].

c-TCD examination: The EMS-9 A TCD monitor (Shenzhen Delikai Electronics Co., Ltd., China) was adopted, and the probe frequency was set at 1.6 MHz. The patient was supine on the examination bed and first underwent a routine TCD examination by a sonographer; the instrument was then set to single-channel dual-depth mode, and the middle cerebral artery was selected from the temporal window for emboli monitoring; if the temporal window was limited, the vertebral artery was selected from the occipital window. Activated saline was bolus injected through the cubital vein under calm breathing conditions, and the presence of microbubble signals was monitored by the sonographer. Next, the patient performed the Valsalva maneuver under the guidance of the sonographer. The patient was instructed to inhale quickly and deeply and to hold his/her breath for 10 s before exhaling. The nurse injected activated saline while the patient held his/her breath. The embolic signals were monitored in real time by the sonographer. A reduction of at least 25% of the mean middle cerebral artery velocity indicated that the Valsalva maneuver was effective [6].

c-TTE examination: Color Doppler ultrasound diagnostic instruments PHILIPS-EPIQ5 and SIEMENSSC2000 were selected and equipped with a 1–5 MHz fan-scan probe. The patient was placed in a left lateral decubitus position. First, routine TTE examination was performed by a sonographer to observe whether there was a fissure in the atrial septum and whether there was a signal of an over septal shunt. Activated saline was then bolus injected through the cubital vein, and the sonographer observed whether there was an RLS microbubble signal in the four-chamber view. Next, the patient performed the Valsalva maneuver under the guidance of the sonographer. The patient was instructed to inhale quickly and deeply and to hold the breath for 10 s before exhaling. The nurse injected activated saline while the patient held his/her breath. The embolic signals were monitored in real time by the sonographer. The Valsalva maneuver was considered effective if the atrial septum protruded into the left atrium after exhalation [8].

Routine monitoring was performed three times: one during calm breathing and two during Valsalva maneuvers. The injections were administered in a space of five minutes. If the inspection result of the first Valsalva maneuver was extensive RLS, a second Valsalva maneuver was not required. If the first two Valsalva maneuvers were invalid, the Valsalva maneuver could be repeated up to five times [14].

TEE examination: This examination was conducted by an experienced sonographer using PHILIPS EPIQ5 color Doppler ultrasound diagnostic instrument, equipped with esophageal probe S7-3t. The patient was informed of the precautions and signed the informed consent form before the examination. The patient slowly swallowed lidocaine hydrochloride mucilage for oropharynx anesthesia five minutes before the examination. The patient was placed in a right lateral decubitus position. After entering the esophagus, the probe was rotated within 90°–120° to clearly display the atrial septum as well as to observe whether an patent foramen ovale or atrial septal defect existed both in two-dimensional and Color Doppler ultrasonography. Foaming test was not added during the TEE inspection in this study.

### Judgment standard

**Fig. 1:**
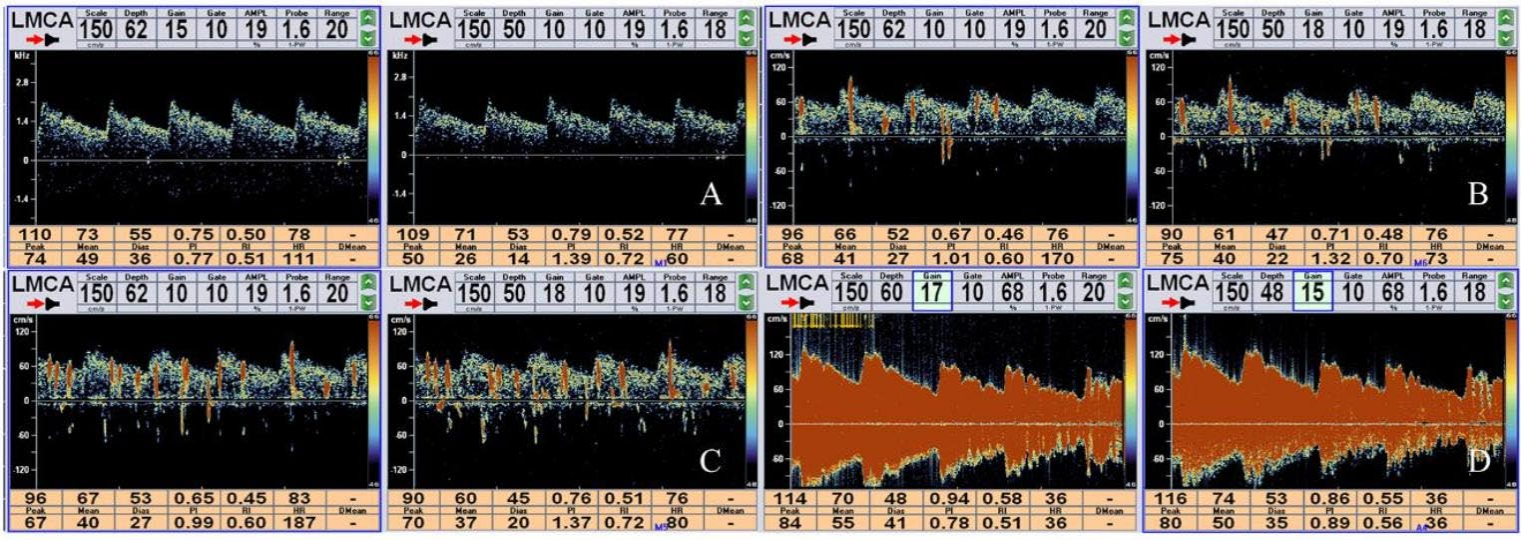
Quantification of RLS by c-TCD No RLS (A), mild RLS (B), moderate RLS (C), and extensive RLS (D) RLS: right-to-left shunt; c-TCD: contrast-enhanced transcranial Doppler

**Fig. 2:**
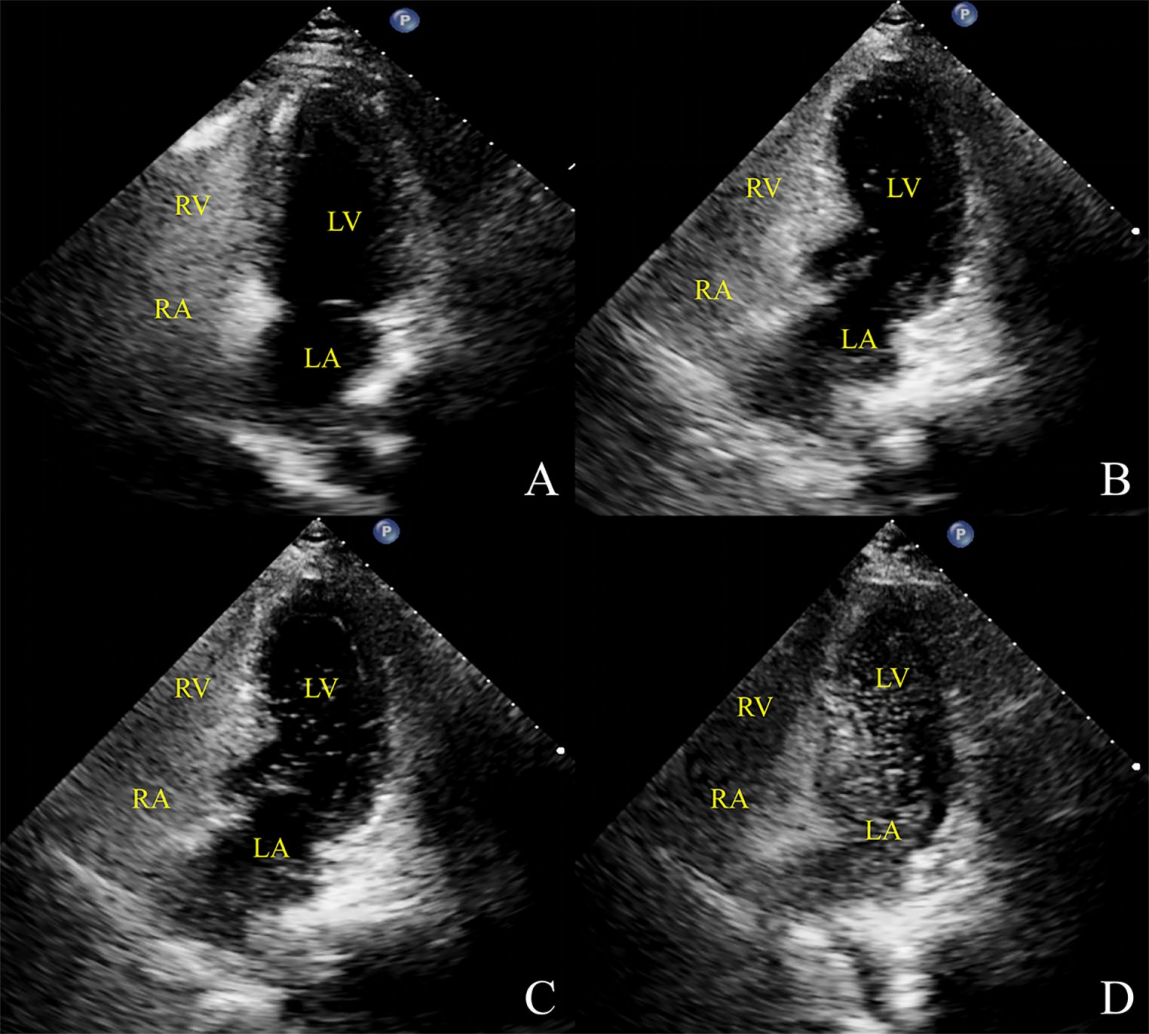
Quantification of RLS by c-TTE No RLS (A), mild RLS (B), moderate RLS (C), and extensive RLS (D) RLS: right-to-left shunt; c-TTE: contrast transthoracic echocardiography

c-TCD was considered positive when the microbubble signal appeared within 25 s. c-TCD was classified according to the number of microbubble signals as follows: negative, no microbubble signal; mild RLS, 1–10 microbubble signals; moderate RLS, 11–25 microbubble signals; extensive RLS, > 25 microbubble signals or rain curtains [6, 15] (Fig. 1).

c-TTE was judged positive based on the presence of a microbubble signal within 10 cardiac cycles. c-TTE was classified according to the number of microbubbles entering the left heart chamber as follows: negative, no microbubble signal; mild RLS, 1–10 microbubbles per frame; moderate RLS, 11–30 microbubbles per frame; extensive RLS, > 30 microbubbles per frame or the left heart chamber filled with microbubbles (Fig. 2) [16].

The size of the shunt flow was determined using the one with the highest microbubble signal in multiple monitoring. The presence of microbubble signals in the resting state is called permanent shunt. If there is no microbubble signal in the resting state, and the microbubble signal appeared after the Valsalva maneuver, it is a provoked shunt.

### Statistical analysis

SPSS Statistics version 26.0 (IBM Corporation, Armonk, NY, USA) was used to perform all statistical analyses. Continuous variables were expressed as mean ± standard deviation. Categorical variables were expressed as frequency percentage. Paired chi-square test was used to test the correlation and dominance of c-TCD and c-TTE. The consistency of the two diagnostic methods was tested using the kappa consistency test. Group I included cases with consistent diagnoses of c-TCD and c-TTE. Group II included cases with inconsistent diagnoses of c-TCD and c-TTE. Group III included patients who were c-TCD(+) and c-TTE(+). Group IV included patients who were c-TCD(+) and c-TTE(−).Group I was compared with Group II. Group III was compared with Group IV. Chi-square test was used to compare sex and shunt mode between groups. Mann-Whitney test was used for age comparison between groups. Differences were considered statistically significant at P < 0.05.

## Results

**Fig. 3:**
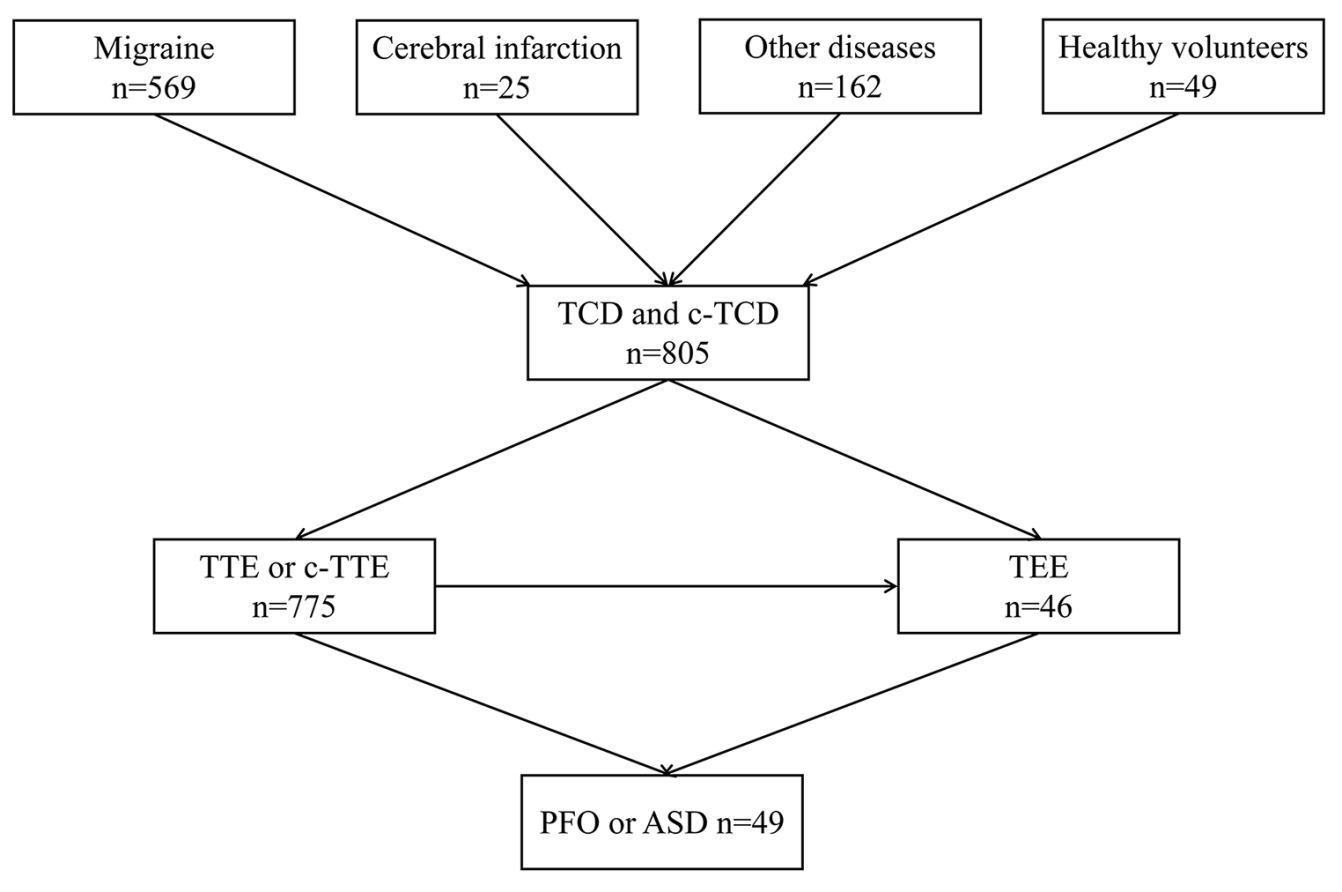
Process of patient examination The 49 cases of PFO or ASD refer to the opening of the foramen ovale or atrial septal defect clearly seen by TTE or TEE. TCD, transcranial Doppler; c-TCD, contrast-enhanced transcranial Doppler; TTE, transthoracic echocardiography; c-TTE, contrast transthoracic echocardiography; TEE, transesophageal echocardiography; PFO, patent foramen ovale; ASD, atrial septal defect

A total of 805 patients were enrolled in this study, all of whom underwent TCD and c-TCD examinations. There were 564 women, with a median age of 43 years (interquartile range: 31–53 years). Among the 805 patients, 569 (71%) were referred for headache, 25 (3%) for cerebral infarction, and 162 (20%) for other diseases; 49 (6%) patients were asymptomatic. A total of 775 and 46 patients, respectively, completed the c-TTE and TEE examinations. A total of 49 patients with PFO or atrial septal defect (ASD) were diagnosed using TTE and TEE. A detailed flowchart is shown in Fig. 3.

A comparison of the results of c-TCD and c-TTE in the diagnosis of RLS is presented in Tables 1 and 693 cases of c-TCD and c-TTE had consistent diagnoses, while 82 cases had inconsistent diagnoses. The diagnostic results of the two methods for RLS had moderate consistency (kappa value = 0.444, P < 0.001). The two diagnostic methods were significantly associated (χ^2^ = 210.24, P < 0.001). The dominance of c-TCD was significantly higher than that of c-TTE (χ^2^ = 78.05, P < 0.001).

**Table 1:** Comparison of diagnostic results between c-TCD and c-TTE

Paired chi-square test		c-TCD		
		+	-	Total
c-TTE	+	654	1	655
	-	81	39	120
	Total	735	40	775
Statistical results	Correlation test		Dominance test	
	χ^2^	P	χ^2^	P
	210.24	< 0.001	78.05	< 0.001

c-TCD: contrast-enhanced transcranial Doppler; c-TTE: contrast transthoracic echocardiography

The 693 cases with consistent diagnoses of c-TCD and c-TTE were set as group I, and the 82 cases with inconsistent diagnoses of c-TCD and c-TTE were set as group II. There was no significant difference in sex or age between groups I and II, but there was a significant difference in the proportion of different shunts (χ^2^ = 80.7, P < 0.001) (Table 2).

**Table 2:** Analysis of the difference between the diagnostic results of c-TCD and c-TTE

	Consistent (Group I) N = 693	Inconsistent (Group II) N = 82	χ^2^/Z	*P*-value
Sex^a^ Male Female	211 (30%) 482 (70%)	20 (24%) 62 (76%)	1.29	0.257
Age^b^	43 (31–53)	46.5 (32–56.5)	-1.757	0.079
No shunt and mild shunt of c-TCD^a^	107 (15.4%)	47 (57.3%)		
Moderate and extensive shunt of c-TCD^a^	586 (84.6%)	35 (42.7%)	80.76	< 0.001

^a^Number (% of total)

^b^Median (interquartile range)

Group I included cases with consistent diagnoses of c-TCD and c-TTE.

Group II included cases with inconsistent diagnoses of c-TCD and c-TTE.

c-TCD: contrast-enhanced transcranial Doppler; c-TTE: contrast transthoracic echocardiography

A total of 654 patients with c-TCD(+) and c-TTE(+) were assigned to group III, and 81 patients with c-TCD(+) and c-TTE(-) were assigned to group IV. There was no significant difference in age or sex between groups III and IV, but there was a significant difference in the proportion of permanent and provoked shunts (χ^2^ = 21.75, P < 0.001) (Table 3).

**Table 3:** Comparison of c-TTE(+) and c-TTE(-) in c-TCD(+)

	c-TCD(+) and c-TTE(+) (Group III) N = 654	c-TCD(+) and c-TTE(-) (Group IV) N = 81	χ^2^/Z	*P-value*
Sex^a^ Male Female	199 (30%) 455 (70%)	19 (23%) 62 (77%)	1.68	0.195
Age^b^	43 (31–53)	47 (32–57)	-1.704	0.088
Permanent shunt of c-TCD^a^	403 (62%)	28 (35%)		
Provoked shunt of c-TCD^a^	251 (38%)	53 (65%)	21.75	< 0.001

^a^Number (% of total)

^b^Median (interquartile range)

Group III included patients who were c-TCD(+) and c-TTE(+).

Group IV included patients who were c-TCD(+) and c-TTE(−).

A total of 49 cases of PFO or ASD were diagnosed by TEE or TTE examination, including 34 women with a median age of 47 years (interquartile range: 36.5–59.5 years). The c-TCD screening results revealed 49 positive cases, including two cases of mild shunt, one case of moderate shunt, and 46 cases of extensive shunt. Further, 44 cases of c-TTE were positive, including 4 cases of mild shunt, 1 case of moderate shunt, and 39 cases of extensive shunt, and the remaining 5 cases were negative. There was no significant difference in the advantages between the two diagnostic methods (χ^2^ = 3.2, P = 0.074).

A total of 46 patients completed both c-TCD and TEE examinations. The c-TCD results were positive in 45 cases and negative in 1 case. TEE revealed PFO in 22 cases, and no septal blood flow was observed in 24 cases. The diagnosis of RLS was inconsistent between the two methods (kappa value = 0.04, P = 0.333).

## Discussion

In our study, c-TCD has the highest sensitivity in diagnosing RLS and is an excellent screening test; however, it cannot differentiate between intracardiac and extracardiac shunts. Although c-TTE and TEE are not as effective as c-TCD in detecting mild RLS, they can help observe intracardiac structure. In the case of obesity and gas interference, TEE is advantageous and can be used as a supplement to c-TTE.

In this study, the test superiority of c-TCD for RLS was significantly greater than that of c-TTE. This result is consistent with the findings of Maillet et al. [11]. Taking c-TCD as a reference, the positive detection rate of c-TTE in the permanent shunt group was significantly higher than that in the provoked shunt group. When the c-TCD and c-TTE results were inconsistent, 99% (81/82) showed c-TCD positivity, and most of them were provoked shunts. We believe that two conditions contribute to the occurrence of RLS: one is the abnormal passage between the arteriovenous system, and the other is that the pressure in the right atrium is higher than that in the left atrium. Under normal conditions, the pressure in the left atrium is higher than that in the right atrium, and no RLS occurs. Coughing, crying, constipation, diving, and Valsalva maneuvers can cause a sudden increase in right atrial pressure and right atrial blood flow, resulting in a higher right atrial pressure than the left atrial pressure, which can lead to RLS. Previous studies have reported that the detection rate of RLS under Valsalva maneuvers is higher than that in the resting state, and Valsalva maneuvers affect the positivity rate of RLS diagnosis [2, 9, 17]. In this study, c-TCD was used to evaluate the effect of Valsalva maneuvers on the degree of cerebral arterial blood flow decline, which can achieve an intuitive and accurate evaluation. However, c-TTE mainly relies on the examiner’s observation of the image to judge the effect of Valsalva maneuvers, which has a certain degree of subjectivity. During c-TTE examination, Valsalva maneuvers were not fully performed in some patients, and some cases were missed due to insufficient increase in right atrial pressure, especially for provoked shunts. This explains why it was difficult to detect shunts with c-TTE when c-TCD was a provoked shunt. Some scholars have proposed that the simultaneous execution of c-TCD and c-TTE can prevent errors caused by ineffective Valsalva maneuvers and improve the detection rate of c-TTE [18]. When the results of c-TCD and c-TTE were inconsistent, only one patient tested negative for c-TCD but positive for c-TTE. This was a mild shunt. It was speculated that the reason may be that the microbubbles passed through undetected blood vessels, which led to a missed diagnosis. The Latin American consensus statement on the use of c-TCD for the diagnosis of RLS suggests that dual-channel monitoring is better than single-channel monitoring [17].

In addition, our study found that the consistency of c-TCD and c-TTE examination results was more common in moderate and extensive shunts, whereas inconsistent results were more common in mild shunts. Literature shows that c-TTE may miss very small PFO [4]. The number of c-TTE microbubbles depends on the clarity of the ultrasound images. Owing to factors such as obesity and gas interference, c-TTE has poor image quality in some patients, and the Valsalva maneuver sometimes leads to further deterioration of image quality, which affects the observation of the number of microbubbles, especially when there are a small number of microbubbles. However, c-TCD was automatically counted by the machine software, and there were almost no cases of missing microbubbles. This resulted in a higher diagnostic sensitivity of c-TCD for RLS than that of c-TTE, especially in mild shunts.

In this study, 49 patients were diagnosed with PFO or ASD, all of whom had a definite defect in the atrial septum on TEE or TTE. In this case, most of the two foaming tests involved moderate or extensive shunting, which also confirmed that the detection rates of c-TCD and c-TTE for moderate and extensive shunting were consistent. This was consistent with the findings of Palazzo et al. [19].

In the past, some scholars believed that TEE was considered to be the gold standard for the detection of PFO [17, 20]. However, more and more studies showed that the detection rate of RLS by TEE was not as good as that of c-TCD [21, 22]. TEE should be performed to accurately assess the morphologies of PFO [9], moreover it is semi-invasive, curborsome, and may cause discomfort to patients, making it less suitable as a first-choice test. The detection rate of TEE for RLS in this study was significantly lower than that of c-TCD, which was consistent with the findings of Mazzucco [21] and Van et al. [12]. The possible reasons for this phenomenon are as follows: first, the foramen ovale was too small to be detected by TEE; second, in the case of provoked shunt, the PFO was always tightly closed at rest; third, there were a few extracardiac shunts, such as pulmonary arteriovenous malformation, in c-TCD-positive patients. Chen et al. found that TEE was more sensitive to RLS only at rest and should not be considered the gold standard [8]. Liu et al. also believed that even with Valsalva maneuvers, c-TEE was less sensitive than c-TTE in detecting PFO [2]. This is because during esophageal intubation, most of the patients’ Valsalva maneuvers could not be performed satisfactorily, resulting in a lower positivity rate of c-TEE than c-TTE or c-TCD.

This study has some limitations. First, this was a retrospective study; second, foaming test was not performed during TEE; third, since most of the c-TCD-negative patients did not undergo further examination, the majority of the patients enrolled in this study were c-TCD-positive, resulting in a selection bias; and fourth, c-TCD could not further differentiate between intracardiac and extracardiac shunts among c-TCD-positive patients. We believe that the simultaneous detection of microvesicles by c-TCD and c-TTE is the direction of future research.

In conclusion, c-TCD was more sensitive than c-TTE in the diagnosis of RLS, especially for the detection of provoked shunts or mild shunts. Therefore, we believe that c-TCD is the preferred screening test for the diagnosis of RLS, and that the combination of c-TTE and TEE can improve the diagnostic accuracy of RLS and provide a basis for treatment plans.
